# RimabotulinumtoxinB in sialorrhea: systematic review of clinical trials

**DOI:** 10.1186/s40734-017-0055-1

**Published:** 2017-06-06

**Authors:** Khashayar Dashtipour, Roongroj Bhidayasiri, Jack J. Chen, Bahman Jabbari, Mark Lew, Diego Torres-Russotto

**Affiliations:** 1 Division of Movement Disorders, Department of Neurology/Movement Disorders, Loma Linda University School of Medicine, Faculty of Medical Offices, 11370 Anderson, Suite B-100, Loma Linda, CA USA; 2 Department of Medicine, Chulalongkorn Center of Excellence for Parkinson’s Disease & Related Disorders, Faculty of Medicine, Chulalongkorn University and King Chulalongkorn Memorial Hospital, Thai Red Cross Society, Bangkok, 10330 Thailand; 3 Department of Rehabilitation Medicine, Juntendo University, Tokyo, Japan; 4 Department of Neurology, Loma Linda University School of Medicine, Loma Linda, CA USA; 5 Loma Linda University, School of Pharmacy, Loma Linda, CA USA; 6 Department of Neurology, Yale University School of Medicine, New Haven, CT USA; 7 Department of Neurology, Keck School of Medicine at the University of Southern California, Los Angeles, CA USA; 8 Department of Neurological Sciences, University of Nebraska Medical Center, Omaha, USA

**Keywords:** Parkinson's disease, Sialorrhea, Drooling, Hypersalivation, Myobloc, Botulinum toxin type B

## Abstract

**Objective:**

The aim of this study was to examine the efficacy, safety and dosing practices of rimabotulinumtoxinB (BoNT-B) for the treatment of patients with sialorrhea based on a systematic review of clinical trials.

**Methods:**

A systematic literature review was performed to identify randomized controlled trials and other comparative clinical studies of BoNT-B for the treatment of sialorrhea published in English between January 1999 and December 2015. Medical literature databases (PubMed, Cochrane Library, and EMBASE) were searched and a total of 41 records were identified. Of these, six primary publications that evaluated BoNT-B for the treatment of sialorrhea met criteria and were included in the final data report.

**Synthesis:**

Total BoNT-B doses ranged from 1500 to 4000 units for sialorrhea. Most of the studies in sialorrhea showed statistically significant benefits of BoNT-B versus placebo (range 4–19.2 weeks). BoNT-B was generally well tolerated across the individual studies; most adverse events reported were considered unrelated to treatment. Adverse events considered potentially associated with BoNT-B included: dry mouth, change in saliva thickness, mild transient dysphagia, mild weakness of chewing and diarrhea.

**Conclusions:**

BoNT-B significantly reduces sialorrhea at doses between 1500 and 4000 units. The relatively mild dose-dependent adverse events suggest both direct and remote toxin effects.

**Electronic supplementary material:**

The online version of this article (doi:10.1186/s40734-017-0055-1) contains supplementary material, which is available to authorized users.

## Introduction

Sialorrhea (or “drooling”) is defined as the overflow of saliva from the mouth caused by excessive production of saliva, the inability to retain saliva within the mouth, or swallowing impairment [1]. It is a common disabling symptom of neurological disorders such as amyotrophic lateral sclerosis (ALS), cerebral palsy (CP) and Parkinsonism [1, 2]. Drooling is observed in 40–80% of patients with advanced stage Parkinson’s disease (PD). In addition, severe sialorrhea is a common, potentially stigmatizing and disabling side-effect of neuroleptic drugs such as clozapine. Next to weight gain and sedation, sialorrhea is a common and frequently stigmatizing side effect occurring in about 30% to 80% of patients receiving clozapine therapy [3, 4].

## Review

Invasive procedures such as parotid excision, duct ligation, and radiation ablation are occasionally used [5–8]. Conservative treatments include oral anticholinergic drugs that block parasympathetic pathways to the salivary gland and inhibit saliva production are often used to treat sialorrhea in these patients [9, 10] but these systemic agents are frequently associated with side effects (cognitive impairment, drowsiness, urinary retention).

Evidence supports BoNT as an effective treatment for drooling with a good tolerability profile [11–15]. Studies of BoNT-B for cervical dystonia have shown a relatively high incidence of dry mouth [16], suggesting that BoNT-B may have a particular predilection for salivary glands [17–19]. Therefore, BoNT-B may be particularly effective in the treatment of secretory disorders compared to Botulinum toxin- type A (BoNT-A) [20–22]. The differences between BoNT-A and BoNT-B with respect to the frequency and severity of dysphagia and dry mouth may be explained by serotype-specific variations in diffusion, cell membrane affinity or systemic spread [23]. However, the specifically greater reduction in salivary gland secretion by BoNT-B has been attributed to an increased affinity of secretory gland cholinergic acceptors for the BoNT-B heavy chain [24].

Though there is data that supports the use of BoNT-B in the treatment of sialorrhea, there are still many questions that remain unanswered regarding dosing, injection techniques, benefits and side effects. The aim of this systematic literature review is to consolidate the knowledge gained in these individual studies with the goal of identifying recommendations that can serve to fill knowledge gaps or at the very least, highlight specific questions to be answered by future research.

## Methodology

The literature search strategy and methods for this systematic review were specified in advance and documented in a protocol. Components of the protocol include the literature search strategy; screening criteria, data extraction methods, and risk for bias appraisal in the studies selected for inclusion (see Additional file 1).

### Screening criteria

Randomized controlled trials (RCTs) and other comparative clinical studies for BoNT-B were included, targeting adult patients with sialorrhea. Primary and secondary efficacy, safety, and dosing endpoints were collected.

#### Literature search strategy and data sources

The literature search strategy was developed using a combination of Medical Subject Headings terms and keywords. Keywords of relevance to the review of sialorrhea were RimabotulinumtoxinB (alternative spellings included “Rimabotulinumtoxin B” OR “rimabotulinum toxin B” OR “Myobloc”), sialorrhea, hypersalivation, drooling and clinical trial. Language (English only) and date limits (January 1999 to December 2015) were also applied. The search was performed in three foundational and comprehensive electronic medical literature databases (PubMed, Cochrane Library, and EMBASE) (Additional file 1). Bibliographic reference lists of systematic reviews identified during screening were searched to identify any relevant studies that were not identified through the electronic database searches.

#### Study selection

**Fig. 1:**
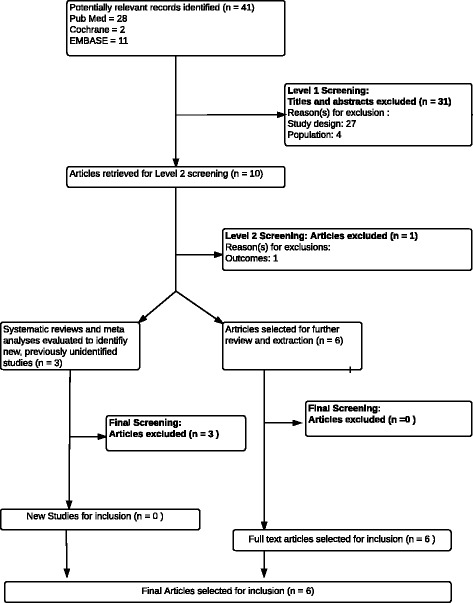
Preferred reporting items for systematic reviews and meta-analyses (PRISMA) flow diagram

At Level 1 screening, all publications reporting preclinical, phase 1, prognostic/biomarker, genetic retrospective, registry, case report and/or non-comparative studies were excluded, as were letters, consensus reports, editorials and nonsystematic reviews. Systematic reviews and meta-analyses were not included but were used to identify additional primary studies. At Level 2 screening, all publications that reported only biochemical or immunologic endpoints were excluded. Also at this stage, nonrandomized controlled phase 2 or 3 clinical trials, comparative long-term follow-up studies (e.g., open-label follow-up of randomized controlled clinical trials), were excluded. Publications reporting secondary and post hoc analyses from a previously published article were not included. The systematic literature review process of study selection was depicted in a Preferred Reporting Items for Systematic Reviews and Meta-Analyses (PRISMA) flow diagram (Fig. 1) [25].

#### Data extraction

Study methodology, patient and treatment level data were extracted from the full-text publications using predefined headings. Each included study underwent quality assessment of risk for bias based on Cochrane metrics. The quality assessment for RCTs systematically addresses six types of bias: selection, performance, detection, attrition, reporting, and other sources of bias not covered by other domains. If non-RCTs or other study types were deemed relevant for data extraction, quality assessment was performed using Transparent Reporting of Evaluations with Nonrandomized Designs appraisal criteria for non-RCTs [26].

## Results

### Publications identified

A total of 41 records were identified from the medical literature databases. Of these, six primary publications that evaluated BoNT-B for the management of sialorrhea were included in the final data report (Fig. 1). Most of the studies fulfilled criteria for low-risk reporting bias. The studies included in the final data report used a wide range of outcome measures, including safety and/or tolerability as assessed by adverse events, Drooling Severity and Frequency Scale (DS-FS [27], visual-analogic ratings of familial distress (VAS-FD) and social distress (VAS-SD) [28], head posture, the Investigator-rated salivation and swallowing items of the Unified Parkinson's Disease Rating (UPDRS) Section II [29] Scale, global impressions of change of illness by the clinician (CGI-Change [30] and subject (PGI-Change), activities of daily living drooling severity scale (DSS), drooling frequency scale (DFS) [31] an adapted version of drool rating scale (DRS) [32]. ALS Functional Rating Scale (ALSFRS-R) [33] Positive and Negative Syndrome Scale (PANSS) Global assessment of functioning (GAF) and Mini Mental State Examination (MMSE) [34] were also assessed as well as salivary gland imaging and objective saliva reduction (saliva production over 5 min using weighing dental rolls).

#### Efficacy in sialorrhea

Given the variability in study design, patient populations, treatment approach, and outcome measures, it is difficult to make comparative statements or general conclusions across the 6 studies included in this review, but there are some similarities that can be highlighted. Total BoNT-B doses ranged between 1500 and 4000 units, with the higher doses showing a trend toward an earlier onset and longer duration of action. The most commonly injected locations were the parotid glands followed by the submandibular gland. It is unclear, based on the studies, if there is a benefit associated with injecting both glands. One study included in this review utilized ultrasound guidance. Though this study showed a statistically significant benefit with a favorable safety profile, it still remains unclear in what way the use of ultrasound guidance influences outcomes. Improvement was noted in several outcome measures, the most notable being visual analog scale (VAS) and Drooling Frequency and Severity Scale (DFSS). No serious AEs were reported. The most commonly reported AE was dry mouth. All of the studies showed statistically significant benefits of BoNT-B in the treatment of sialorrhea versus placebo.

Table 1 provides an overview of the efficacy and safety outcomes from each of the studies.

**Table 1:** Completed Trials for Sialorrhea

Study	Design/Duration	Objective	N	Patient Population	Intervention	Glands injected	Efficacy Outcomes	Safety Outcomes
Chinnapongse R, et al; 2012 [24]	Multicenter, randomized, double-blind, sequential-dose escalation design	Evaluate safety, tolerability and efficacy of three BoNT-B doses subjects with sialorrhea	*N* = 54	PD subjects with troublesome sialorrhea	BoNT-B doses: 1500 units; 2500 units; or 3500 units or placebo	PG and SMG	4 weeks post injection-significant improvement in DFSS and USFR. placebo (p ≤ 0.0009)	No serious AEs attributed to BoNT-B
Guidubaldi A, et al; 2011 [25]	Prospective, randomized, double-blind, crossover pilot study	Evaluate efficacy and tolerability of BoNT-A versus BoNT-B for the treatment of sialorrhea	*N* = 27	Patients with ALS or PD (*n* = 15 ALS; *n* = 12 PD) enrolled,	250 units BoNT-A 2500 units BoNT-B Ultrasound-guided injections into parotid and submandibular glands performed	B/L PG and SMG	Subjective and objective improvements in all patients Shorter latency to response with BoNT-B Similar mean benefit duration similar	Only toxin-related side effect was a change in saliva thickness
Jackson CE, et all; 2009 [26]	Double-blind, randomized study	Determine patient perception of benefit of BoNT-B treatment for sialorrhea in ALS patients	*N* = 20	ALS patients with sialorrhea refractory to medical therapy	2500 units of BoNT-B	B/L PG and SMG	Global impression of improvement of 82% at 2 weeks vs. 38% placebo group (*p* < 0.05) Significant effect sustained at 4 weeks, with continued improvement at 12 wees (in 50% of patients)	No significant AEs (including dysphagia) reported
Ondo, et al; 2004 [27]	Double-blind placebo-controlled	Determine whether BoNT-B is safe and effective for sialorrhea in patients with PD	*N* = 16	PD subjects with problematic sialorrhea	1000 units BoNT-B into each parotid gland and 250 units into each submandibular gland) or placebo	PG and SMG	Improvement on the Visual Analogue Scale,global impressions of change, Drooling Rating Scale and DSFS in BoNT-B	Adverse events were considered mild
Lagalla G, et al; 2009 [28]	Double-blind, randomized, placebo-controlled study	Investigate safety, efficacy and effectiveness of BoNT-B into the parotid glands to reduce drooling in PD subjects	*N* = 36	Advanced phase PD subjects who complained of disabling drooling	4000 units BoNT-B or placebo	PG	BTX-B patients showed a significant improvement in almost all subjective outcomes All BTX-B subjects reported sialorrhea reduction (moderate in 44.4%, and marked in 33.3%), vs 61.1% controls who denied benefit Benefits lasted on average 19.2 +/- 6.3 weeks in the BTX-B)	Three BoNT-B patients complained of mild, transient swallowing difficulties starting 10 days after the injections and recovering within 2 weeks One BoNT-B patient showed a transient mild weakness of chewing.
Steinlechner, et al; 2010 [29]	Double-blind placebo-controlled Patients were followed over 16 weeks	To evaluate the treatment effects, tolerance, and duration of BoNT-B in neuroleptic-induced (group 1) and PD-associated sialorrhea (group 2)	*N* = 9	Group 1: 4 patients Group 2: 5 patients	2500 units BoNT-B) injected under ultrasound control	PG and SMG	“Large effect sizes* for improvement of sialorrhea” in patients treated with BoNT-B compared to placebo. Reduction of sialorrhea lasted for 8 to 16 weeks after a single injection.	No patient reported side effects.

*AE* adverse event, *ALS* amyotrophic lateral sclerosis, *B/L* bilateral, *BoNT-A* abotulinumtoxinA, *BoNT-B* botulinum neurotoxin type B (rimabotulinumtoxinB), *DSFS* Drooling Severity and Frequency Scale, *GI* gastrointestinal, *PD* Parkinson’s disease, *PG* parotid gland, *SMG* submandibular gland, *UPDRS* Unified Parkinson's Disease Rating Scale, *VAS-FD* visual-analogic ratings of familial distress, *VAS-SD* social distress. * Effect size estimates of key measures as a descriptive approach using Cohen's d procedure were applied to measure the magnitude of the treatment effect. Notably, these estimates are independent of sample size unlike significance tests. Small effect size of ≥0.2 and <0.5. Medium effect size of ≥0.5 and <0.8. *Large effect size of ≥0.8. Positive effect sizes implying a decrease of test value from T0 to T1, whereas negative effect sizes implying an increase of test value from T0 to T1, respectively

Chinnapongse and colleagues [35] designed a multicenter, randomized, double-blind, sequential-dose escalation study. This study included 54 PD subjects with troublesome sialorrhea. Each subject was randomized to receive only 1 treatment with a BoNT-B dose (1500, 2500, or 3500 Units) or matched placebo. Study medication was injected first into the submandibular glands, followed by the parotid glands. The dose for the submandibular glands for all subjects receiving BoNT-B was 250 Units per gland. The doses for the parotid glands were 500, 1000, or 1500 Units per gland for the 1500, 2500, and 3500 Units groups, with volume matched placebo groups, respectively. Injections were guided solely by external anatomical landmarks following a standardized procedure. Subjects were contacted by telephone approximately 24 h post injection to regarding their health status, they then returned for follow-up assessments at weeks 1, 2, 4, 8, 12, 16, and 20 post injection. Subjects also reported the DFSS and salivation item of a subject-report UPDRS Section II daily using an electronic diary. Safety and tolerability (assessed by adverse events) was the primary outcome measure. Efficacy (assessed by the DFSS and unstimulated salivary flow rate (USFR)) was the secondary outcome. Gastrointestinal-related adverse events occurred more frequently in the active groups versus placebo group (31% vs. 7%), with dry mouth being most common (15%). There were no BoNT-B related serious adverse events or discontinuations. At 4 weeks post injection, there was a dose-related significant improvement in DFSS and USFR significantly decreased in all active groups versus placebo. The authors stated that treated subjects appeared to have more sustained improvement in sialorrhea. The authors concluded that intraglandular injections of BoNT-B were safe, tolerable and efficacious for the treatment of sialorrhea in PD patients. They further stated that a subsequent phase 3 clinical trial to further confirm the drug’s robust efficacy has recently completed recruitment and the results are pending (identified as clinicaltrials.gov identifier, NCT01994109).

Guidubaldi et al [36] reported on a study of consecutive ALS and PD patients affected by severe sialorrhea randomized to receive BoNT-A or BoNT-B injections into the salivary glands. When sialorrhea returned to baseline (at least 3 months after the first injection) following the first treatment, subjects were re-treated with the other serotype. Ultrasound (US)-guided BoNT injections into each parotid gland (two sites per gland) and each submandibular gland (one site per gland) were performed bilaterally by the same physician. The injections were performed under continuous US guidance. Total doses were 250 units BoNT-A and 2500 units BoNT-B. 2500 U of BoNT-B was used based on previous reports. Objective (cotton roll weight) and subjective (ad hoc clinical scales) evaluations were performed at baseline, after 1 and 4 weeks, and every 4 weeks until drooling returned to baseline.. The primary endpoint was the magnitude of change to the weight of the cotton rolls. Secondary endpoints included: DSS (range: 0–4), DFS (range 0–3), an adapted version of (range0–45), a VAS range 0–10), and a CGIC range2–13). The secondary endpoints were designed to show the impact of drooling on daily life. The safety assessment was based on reported side effects. Twenty-seven patients (15 ALS and 12 PD) were enrolled and 14 patients completed the study. Thirteen patients (mean age 71.5 -7.4) were lost at follow-up after the first treatment (due to deaths not related to BoNT and inability to respect the follow-up visits schedule due to the advanced stage of disease). No patient abandoned the study because of side effects. The authors found that BoNT-A and BoNT-B treatments gave both subjective and objective improvements in all patients. They stated that the latency was significantly shorter after BoNT-B treatments compared to BoNT-A. The mean benefit duration was similar at 75 and 90 days for BoNT-A and BoNT-B, respectively. The authors reported change in saliva thickness as the only toxin-related side effect. The authors concluded that either 250 units BoNT-A or 2500 units BoNT-B had similar efficacy and safety in the treatment of sialorrhea. BoNT-B was shown to have a shorter latency and comparable duration and stated that cost analysis, based on the doses used in this study, favored BoNT-B treatment.

Jackson and colleagues [37] reported the results of a double-blind, randomized study of twenty ALS patients with sialorrhea refractory to medical therapy. The subjects received either 2500 units of BoNT-B or placebo into the bilateral parotid and submandibular glands using electromyographic guidance. A total of eight injections (two per gland bilaterally) were performed into both the parotid and submandibular glands. Electromyography was used to aid in the placement of the needle and to avoid intramuscular injection. Outcome assessments were performed at weeks 2, 4, 8, and 12. Medication changes and adverse events were also recorded at these time points. At the 12 week visit a physical examination was performed, and a final global impression of change was performed by the subject, the caregiver, and the investigator. Assessments were performed at baseline (day of injection) and at all follow-up visits (2, 4, 8, and 12 weeks after injection). The ALS Functional Rating Scale was performed by phone 1 week after the baseline visit. If there was more than a 1 point change (denoting deterioration in function) on either the swallowing question or the respiratory questions (dyspnea, orthopnea, respiratory insufficiency), the patient visited with the investigator. Subjective outcome measurements were recorded prior to interaction with the study physician. The authors found that patients who received BoNT-B reported a global impression of improvement of 82% at 2 weeks compared to 38% of those who received placebo. This significant effect was sustained at 4 weeks. At 12 weeks, 50% of patients who received BoNT-B continued to report improvement compared to 14% of those who received placebo. There were no significant adverse events, including dysphagia, in the BoNT-B group.

Ondo et al [38] reported the results of a study designed to determine the safety and efficacy of BoNT-B for the treatment of sialorrhea in patients with PD. Demographics, PD treatments, head posture, the UPDRS, two questionnaires regarding drooling, VAS, global impressions, salivary gland imaging and a dysphagia questionnaire were assessed in 16 PD subjects with problematic sialorrhea. Patients were then randomized (1:1 ratio) to receive either BoNT-B or a pH-matched vehicle. A total of 2500 units was injected (1000 divided at two sites into each parotid gland and 250 at one site into each submandibular gland). All injection sites were localized with anatomic markers All patients returned 1 month later and repeated the assessment. The patients were offered a single open-label injection at that visit or at a later time at their request Compared with placebo, those randomized to drug reported improvement on the VAS, global impressions of change, DRS and DSFS. The authors reported no change in UPDRS, head posture, or Dysphagia Scale. Adverse events were mild and included dry mouth (three patients), worsened gait (two patients), diarrhea (one patient), and neck pain (one patient) in the BoNT-B group. The authors stated that anatomically guided injections of BoNT-B into the parotid and submandibular glands appeared to effectively improve sialorrhea without causing dysphagia in patients with PD.

Lagalla et al [39] investigated the safety, efficacy and effectiveness of BoNT-B injections into the parotid glands to reduce drooling in PD subjects in a double-blind, randomized, placebo-controlled study that enrolled 36 advanced phase PD subjects who complained of disabling drooling. Patients received either 4000 units BoNT-B or placebo. Anatomically guided injections were performed. Outcome measures were chosen to assess both the subjective feeling of improvement (i.e. the DSFS, VAS-FD, VAS-SD) and objective saliva reduction (saliva production over 5 min assessed by weighing dental rolls). The GIS was also applied, rating improvement from 0 to 3. A comprehensive clinical assessment was performed at baseline and 1 month after treatment. Subsequently, on a monthly schedule, telephone calls were performed to ask patients about the persistence of benefit. The impact of drooling on daily life was checked using the Drooling Severity and Frequency Scale (DS-FS), as well as VAS-FD and VAS-SD). All the quoted measures required rating symptom severity from 0 (no disability/distress) to 100 (maximum disability/distress ever experienced). The UPDRS-ADL item scores were also recorded for drooling and swallowing (dysphagia). When assessed 1 month after injections, BoNT-B patients showed a meaningful improvement in almost all subjective outcomes. All BoNT-B subjects reported sialorrhea reduction (moderate in 44.4%, marked in 33.3%), compared with 61.1% of controls, who reported no benefits. Benefits lasted on average of 19.2 ± 6.3 weeks in the BoNT-B group compared to 6.7 ± 1.4 weeks in the control group (T value: 26.4; *p* < 0.0001). The authors concluded that BoNT-B injections were safe and effective in the management of PD-related drooling.

Steinlechner et al [40] reported the results of a 16-week, double-blind, placebo-controlled trial designed to evaluate the treatment effects, tolerance and duration of BoNT-B injections in the treatment of severe sialorrhea due to neuroleptics (*N* = 4) or associated with PD (*N* = 5). Patients were randomized to receive injections of either BoNT-B or sodium chloride as placebo. A total of either 2500 units BONT-B (500 units into each parotid gland and 250 units or 0.5 ml 0.9% sodium chloride was injected into each submandibular gland) under ultrasound guidance. All patients completed the motor portion of the UPDRS (part III), the Positive and Negative Syndrome Scale (PANSS), and answered validated questionnaires regarding their subjective drooling problems, including the DSFS. Quantitative saliva measurements (including saliva weight and evaluation of saliva concentration of albumin, total protein, and immune globulins A, G, and M) were conducted prior to the injections and at a follow-up visit 4 weeks after the injections. Saliva weight was measured using six dental rollsSaliva concentrations of albumin, total protein, and immunoglobulins A, G, and M were determined to demonstrate the efficacy of the BONT-B treatment. The authors stated that when compared with the placebo group at 4 weeks post injection, the BONT-B group showed a significant reduction in saliva weight. No patient reported adverse events. Reduction of sialorrhea lasted for 8 to 16 weeks after a single injection. The authors concluded that similar to what has been observed in PD; BoNT-B represented an effective and safe treatment for neuroleptic-induced sialorrhea.

#### Safety

BoNT-B was generally well tolerated across all studies. Most adverse events reported were considered unrelated to treatment. No treatment-related serious adverse events were reported. Adverse events considered potentially associated with BoNT-B included: gastrointestinal-related adverse events, dry mouth, and change in saliva thickness, mild transient swallowing difficulties, and transient mild weakness of chewing, worsened gait, diarrhea and neck pain. There is no clear evidence that the side effect profile is related to patient population (etiology, disease severity) or injection technique.

## Discussion

Sialorrhea negatively affects patients’ quality of life, interfering with social participation and increasing care burden [41]. To date, treatment options have been aimed at reducing the unfavorable impact of drooling on social interaction and decreasing the risk of aspiration-related lung infections. The data we presented here show the efficacy of BoNT-B, when compared with other BoNTs and placebo, with adequate tolerability. In the *2009 Evidence-based Guideline for Clinicians- The Care of the Patient with Amyotrophic Lateral Sclerosis: Multidisciplinary Care, Symptom Management, and Cognitive/Behavioral Impairment*, the American Academy of Neurology concluded that there is good evidence in patients with ALS who have medically refractory sialorrhea and that BoNT-B should be considered (Level B evidence) [42]. Naumann and colleagues reviewed four Class II studies in the treatment of sialorrhea in Parkinson’s disease (3 BoNT-A and 1 BoNT-B) and concluded that BoNT is probably safe and effective for the treatment drooling in patients with PD (four Class II studies), BoNT should be considered as a treatment option for palmar hyperhidrosis and drooling (Level B) [43]. Lakraj and colleagues evaluated the level of evidence for BoNT efficacy based on reviewed studies. The authors stated that in adults, the level of evidence for BoNT-B is A (established efficacy), based on 2 class I, 3 class II and 1 class III studies [44]. Narayanaswami [45] recently published the results of a meta-analysis of six studies that demonstrated significant benefit of Botulinum toxin on functional outcomes.

As previously mentioned, there are many studies that have been designed to evaluate the efficacy and safety of BoNT in the treatment of sialorrhea. Based on the criteria for conducting a systematic literature review, many of those studies were excluded (and therefore not discussed here), though the studies that are presented here provide a representation of the information garnered from the excluded studies. In the absence of data from a large-well-controlled study designed to specifically examine these parameters, it is still difficult to fill many of the knowledge gaps in this area. Most published studies on BoNT in sialorrhea were non-blinded and uncontrolled, and hence may be subjected to biased interpretation. These studies frequently involved small numbers of patients and therefore may lack statistical power for meaningful analysis. Dose-ranging information was frequently not available, so the optimal BoNT dose is not clear from these studies. The relative superiority of injecting two glands (e.g. parotid and submandibular) over a single gland has not been clarified. The ideal sites of gland injections and the added value of ultrasound guidance still needs to be further examined. Proponents for blind injection argue that anatomic landmarks of the salivary glands are easy to locate, the cholinergic innervation is equally distributed throughout the glands and there is no specific or optimal site to target with ultrasound. Those who support ultrasound guidance suggest that the technique guarantees precise and safe delivery of BoNT into the glands especially if there are anatomic variations, and it allows a more accurate documentation of glandular alteration after injection. Furthermore in some elderly patients, the salivary glands may be atrophied, making localization difficult. Investigators have used varying outcome measures to analyze sialorrhea, ranging from visual analogue scales to counting of dental rolls to clinical rating scales, and this makes comparison between some studies difficult. This is especially true based on the difficulty of quantification of saliva, as its production may vary with time of the day. In the studies reviewed here that utilized objective measures), a clear benefit was observed with the use of BoNT-B [30, 33, 34]. Again, given the differences in doses used and glands injected, it is difficult to make a comparative statement regarding the studies. Clinical experience and skill of the investigators as a potential confounding factor on the outcome has not been evaluated. A true cost effective analysis of BoNT compared to the best medical treatment is not available, while the relative efficacy of BoNT-A versus BoNT-B is unclear and outcome data on efficacy and adverse effects from prospective, long-term studies are still lacking.

### Limitations

In this systematic review, a quality assessment that included the risk for bias criteria presented in the Cochrane Handbook for Systematic Reviews of Interventions Version 5.1.024 [46] was applied. This resulted in the exclusion of large uncontrolled studies and other studies that did not meet the predefined assessment criteria, which may have precluded the use of relevant data. This also led to the inclusion (in some cases) of studies that may have been excluded from other evidence-based reviews, based on sample size. Further, most of the applicable studies applied only a single set of injections with no or only short-term follow-up. Importantly, the endpoints varied and were comprised of both subjective and objective measurements. Nevertheless, subjective endpoints concurred with objective measurements of salivary flow reduction, as measured by weighing saliva-soaked dental rolls. A possible methodological limitation of trials in patients with sialorrhea arises from the lack of a validated outcome measure. This is further complicated by inherent inconsistency when using subjective endpoints. There is no consensus regarding the currently used scales including the drooling severity scale and drooling frequency scale. Examples of this are seen throughout the literature (for example: Mancini [47] considered a 2-point change was clinically significant, whereas Lipp [48] showed that objective and subjective measures of drooling are not necessarily in agreement. Given this, future studies should explore and validate new clinical scales for sialorrhea and define clinically meaningful outcome measures. It is also important to note that these studies were all short term, usually the result of a single injection session, and as such, they offer no information about long-term safety and efficacy of BoNT-B for treatment of sialorrhea. The influence that the use of atypical neuroleptics in the treatment of these patients may have had on the presentation of sialorrhea seen in studies presented here, is also an important consideration.

## Conclusions and perspectives

On the basis of data extracted from six qualifying randomized clinical studies, there is strong evidence for the safety and efficacy of BoNT-B in the treatment of sialorrhea. Though the studies reviewed here provide valuable information, many unanswered questions remain. A topic that is frequently raised is the use of ultrasound guidance and whether it provides a benefit in regards to safety and efficacy. There is still uncertainty about the best starting dose and dose range as well as which glands should be injected (i.e. Do we always need to inject both the parotid and the submandibular?). Whether a differential benefit is seen based on etiology (PD, CP, ALS) is also a topic that is often raised. These questions are not clearly answered in the current literature. It is our hope that future studies will provide some of these answers. The recently completed phase 3 clinical trial program for BoNT-B for the treatment of sialorrhea (clinicaltrials.gov identifier, NCT01994109) may serve to provide further evidence for this indication and begin to answer some of these and other previously unanswered questions.

## Additional file

Additional file 1: Systematic Literature Review of RimabotulinumtoxinB in Clinical Trials for Parkinson’s disease. (PDF 502 kb)

## Abbreviations

ALS: Amyotrophic lateral sclerosis
ALSFRS-R: ALS Functional Rating Scale
BONT-A: Botulinum toxin- type A
BoNT-B: rimabotulinumtoxinB
CGI: C Global impressions of change of illness by the clinician
CP: Cerebral palsy
DFS: Drooling frequency scale
DFSS: Drooling Frequency and Severity Scale
DRS: Drool rating scale
DS-FS: Drooling Severity and Frequency Scale
DS-FS: Drooling Severity and Frequency Scale
DSS: Drooling severity scale
GAF: Global assessment of functioning
MMSE: Mini Mental State Examination
PANSS: Positive and Negative Syndrome Scale
PANSS: Positive and Negative Syndrome Scale
PD: Parkinson’s disease
PGI-Change: Patient Global Impressions of Change of Illness
PRISMA: Preferred Reporting Items for Systematic Reviews and Meta-Analyses
RCTs: Randomized controlled trials
UPDRS: Unified Parkinson's disease Rating
US: Ultrasound
USFR: Unstimulated Salivary Flow Rate
VAS: Visual analog scale
VAS-FD: Visual-analogic scale of familial distress
VAS-SD: Visual-analogic scale and social distress

## References

[1] Meningaud JP, Pitak-Arnnop P, Chikhani L, Bertrand JC (2006). Drooling of saliva: a review of the etiology and management options. Oral Surg Oral Med Oral Pathol Oral Radiol Endod.

[2] Giess R, Naumann M, Werner E, Riemann R, Beck M, Puls I, Reiners C, Toyka KV (2000). Injections of botulinum toxin A into the salivary glands improve sialorrhea in amyotrophic lateral sclerosis. J Neurol Neurosurg Psychiatry.

[3] Ben-Aryeh H, Jungerman T, Szargel R, Klein E, Laufer D (1996). Salivary flow-rate and composition in schizophrenic patients on clozapine: subjective reports and laboratory data. Biol Psychiatry.

[4] Davydov L, Botts SR (2000). Clozapine-induced hypersalivation. Ann Pharmacother.

[5] Andersen PM, Gronberg H, Franzen L, Funegard U (2001). External radiation of the parotid glands significantly reduces drooling in patients with motor neurone disease with bulbar paresis. J Neurol Sci.

[6] Ethunandan M, Macpherson DW (1998). Persistent drooling: treatment by bilateral submandibular duct transposition and simultaneous sublingual gland excision. Ann R Coll Surg Engl.

[7] Stern Y, Feinmesser R, Collins M, Shott SR, Cotton RT (2002). Bilateral submandibular gland excision with parotid duct ligation for treatment of sialorrhea in children: long-term results. Arch Otolaryngol Head Neck Surg.

[8] Mankarious LA, Bottrill ID, Huchzermeyer PM, Bailey CM (1999). Long-term follow-up of submandibular duct rerouting for the treatment of sialorrhea in the pediatric population. Otolaryngol Head Neck Surg.

[9] Bagheri H, Damase–Michel C, Lapeyre–Mestre M, Cismondo S, O'Connell D, Senard JM, Rascol O, Montastruc JL (1999). A study of salivary secretion in Parkinson’s disease. Clin Neuropharmacol.

[10] Johnston BT, Li Q, Castell JA, Castell DO (1995). Swallowing and esophageal function in Parkinson’s disease. Am J Gastroenterol.

[11] Bhatia KP, Munchau A, Brown P (1999). Botulinum toxin is a useful treatment in excessive drooling in saliva. J Neurol Neurosurg Psychiatry.

[12] Pal PK, Calne DB, Calne S, Tsui JK (2000). Botulinum toxin A as treatment for drooling saliva in PD. Neurology.

[13] Porta M, Gamba M, Bertacchi G, Vaj P (2001). Treatment of sialorrhoea with ultrasound guided botulinum toxin type A injection in patients with neurological disorders. J Neurol Neurosurg Psychiatry.

[14] Racette BA, Good L, Sagitto S, Perlmutter JS (2003). Botulinum toxin B reduces sialorrhea in parkinsonism. Mov Disord.

[15] Contarino MF, Pompili M, Tittoto P, Vanacore N, Sabatelli M, Cedrone A, Rapaccini GL, Gasbarrini G, Tonali PA, Bentivoglio AR (2007). Botulinum toxin B ultrasound-guided injections for sialorrhea in amyotrophic lateral sclerosis and Parkinson’s disease. Parkinsonism Relat Disord.

[16] Brin MF, Lew MF, Adler CH, Comella CL, Factor SA, Jankovic J, O'Brien C, Murray JJ, Wallace JD, Willmer-Hulme A, Koller M (1999). Safety and efficacy of NeuroBloc (botulinum toxin type B) in type A-resistant cervical dystonia. Neurology.

[17] Lang AM (2003). A preliminary comparison of the efficacy and tolerability of botulinum toxin serotypes A and B in the treatment of myofascial pain syndrome: a retrospective, open-label chart review. Clin Ther.

[18] Comella CL, Jankovic J, Shannon KM, Tsui J, Swenson M, Leurgans S, Fan W, Dystonia Study Group (2005). Comparison of botulinum toxin serotypes A and B for the treatment of cervical dystonia. Neurology.

[19] Tintner R, Gross R, Winzer UF, Smalky KA, Jankovic J (2005). Autonomic function after botulinum toxin type A or B: a double-blind, randomized trial. Neurology.

[20] Baumann LS, Halem ML (2003). Systemic adverse effects after botulinum toxin type B (myobloc) injections for the treatment of palmar hyperhidrosis. Arch Dermatol.

[21] Dressler D, Benecke R (2003). Autonomic side effects of botulinum toxin type B treatment of cervical dystonia and hyperhidrosis. Eur Neurol.

[22] Dressler D, Benecke R (2004). Autonomic side effects of botulinum toxin type B therapy. Adv Neurol.

[23] Ramirez-Castaneda J, Jankovic J, Comella C, Dashtipour K, Fernandez HH, Mari Z (2013). Diffusion, spread, and migration of botulinum toxin. Mov Disord.

[24] Gautam D, Heard TS, Cui Y, Miller G, Bloodworth L, Wess J. Cholinergic stimulation of salivary secretion studied with M1 and M3 muscarinic receptor single- and double-knockout mice. Mol Pharmacol. 2004;66(2):260–267. 10.1124/mol.66.2.260 15266016

[25] Moher D, Liberati A, Tetzlaff J, Altman DG, The PRISMA Group (2009). Preferred Reporting Items for Systematic Reviews and Meta-Analyses: The PRISMA Statement. BMJ.

[26] Des Jarlais DC, Lyles C, Crepaz N, TREND Group (2004). Improving the reporting quality of nonrandomized evaluations of behavioral and public health interventions: the TREND statement. Am J Public Health.

[27] Evatt ML, Chaudhuri KR, Chou KL, Cubo E, Hinson V, Kompoliti K, Yang C, Poewe W, Rascol O, Sampaio C, Stebbins GT, Goetz CG (2009). Dysautonomia rating scales in Parkinson’s disease: sialorrhea, dysphagia, and constipation critique and recommendations by movement disorders task force on rating scales for Parkinson’s disease. Mov Disord.

[28] Thomee R, Grimby G, Wright BD, Linacre JM (1995). Rasch analysis of Visual Analog Scale measurements before and after treatment of Patellofemoral Pain Syndrome in women. Scand J Rehabil Med.

[29] Fahn S, Elton RL, UPDRS program members. Unified Parkinsons Disease Rating Scale. In: Fahn S, Marsden CD, Goldstein M, Calne DB, eds. Recent developments in Parkinsons disease, Vol 2. Florham Park, NJ: Macmillan Healthcare Information.1987. p.153–163.

[30] Guy W. Clinical Global Impressions (CGI). In: ECDEU Assessment Manual for Psychopharmacology. Rockville, MD: US Department of Health and Human Services, Public Health Service, Alcohol Drug Abuse and Mental Health Administration, NIMH Psychopharmacology Research Branch; 1976.218–222.

[31] Heine RG, Catto-Smith AG, Reddihough DS (1996). Effect of antireflux medication on salivary drooling in children with cerebral palsy. Dev Med Child Neurol.

[32] Suskind DL, Tilton A (2002). Clinical study of botulinum-A toxin in the treatment of sialorrhea in children with cerebral palsy. Laryngoscope.

[33] Cedarbaum JM, Stambler N, Malta E, Fuller C, Hilt D, Thurmond B, Nakanishi A (1999). The ALSFRS-R: a revised ALS functional rating scale that incorporates assessments of respiratory function. BDNF ALS Study Group (Phase III). J Neurol Sci.

[34] Rapp D (1980). Drool control: long-term follow-up. Dev Med Child Neurol.

[35] Chinnapongse R, Gullo K, Nemeth P, Zhang Y, Griggs L (2012). Safety and efficacy of botulinum toxin type B for treatment of sialorrhea in Parkinson's disease: a prospective double-blind trial. Mov Disord.

[36] Guidubaldi A, Fasano A, Ialongo T, Piano C, Pompili M, Masciana R, Siciliani L, Sabatelli M, Bentivoglio A (2011). Botulinum toxin A versus B in sialorrhea: a prospective, randomized, double-blind crossover pilot study in patients with amyotrophic lateral sclerosis or Parkinson’s disease. Mov Disord.

[37] Jackson CE, Gronseth G, Rosenfeld J, Dubinsky R, Simpson CB, McVey A, Kittrell PP, Herbelin L, Muscle and Study Group (2009). Randomized double-blind study of botulinum toxin type B for sialorrhea in ALS patients. Muscle Nerve.

[38] Ondo WG, Hunter C, Moore W (2004). A double-blind placebo-controlled trial of botulinum toxin B for sialorrhea in Parkinson’s disease. Neurology.

[39] Lagalla G, Millevolte M, Capecci M, Provinciali L, Ceravolo M (2009). Long-lasting benefits of botulinum toxin type B in Parkinson’s disease-related drooling. J Neurol.

[40] Steinlechner S, Klein C, Moser A, Lencer R, Hagenah J (2010). Botulinum toxin B as an effective and safe treatment for neuroleptic-induced sialorrhea. Psychopharmacology (Berl).

[41] Bateson MC, Gibberd FB, Wilson RS (1973). Salivary symptoms in Parkinson’s disease. Arch Neurol.

[42] Miller RG, Jackson CE, Kasarskis EJ, England JD, Forshew D, Johnston W, Kalra S, Katz JS, Shoesmith C, Strong MJ, Woolley SC, Quality Standards Subcommittee of the American Academy of Neurology (2009). Practice, Parameter update: The care of the patient with amyotrophic lateral sclerosis: multidisciplinary care, symptom management, and cognitive/behavioral impairment (an evidence-based review): report of the Quality Standards Subcommittee of the American Academy of Neurology. Neurology.

[43] Naumann M, So Y, Argoff CE, Childers MK, Dykstra DD, Gronseth GS, Jabbari B, Kaufmann HC, Schurch B, Silberstein SD, Simpson DM (2008). Therapeutics and Technology Assessment Subcommittee of the American Academy of Neurology. Neurology.

[44] Lakraj AA, Moghimi N, Jabbari B (2013). Sialorrhea: anatomy, pathophysiology and treatment with emphasis on the role of botulinum toxins. Toxins (Basel).

[45] Narayanaswami, Pushpa et al; Drooling in Parkinson’s disease: A randomized controlled trial of incobotulinum toxin A and meta-analysis of Botulinum toxins. Parkinsonism Relat Disord. 2016;30:73-77. 10.1016/j.parkreldis.2016.07.001 27406786

[46] Higgins JPT, Green S (eds): Cochrane Handbook for Systematic Reviews of Interventions Version 5.1.0 [updated March 2011]. The Cochrane Collaboration, 2011. Available from http://handbook.cochrane.org/. Assessed 2 Dec 2016.

[47] Lipp A, Trottenberg T, Schink T, Kupsch A, Arnold G (2003). A randomized trial of botulinum toxin A for treatment of drooling. Neurology.

[48] Mancini F, Zangaglia R, Cristina S, Sommaruga MG, Martignoni E, Nappi G, Pacchetti C (2003). Double-blind, placebo-controlled study to evaluate the efficacy and safety of botulinum toxin type A in the treatment of drooling in parkinsonism. Mov Disord.

