# Supplementary material for: RimabotulinumtoxinB in sialorrhea: systematic review of clinical trials
Source: J Clin Mov Disord. 2017 Jun 6;4:9. doi: 10.1186/s40734-017-0055-1 (PMC5460542; doi:10.1186/s40734-017-0055-1)
Supplement: Additional file 1: — Systematic Literature Review of RimabotulinumtoxinB in Clinical Trials for Parkinson’s disease. (PDF 502 kb) [file 40734_2017_55_MOESM1_ESM.pdf]

Systematic Literature Review of RimabotulinumtoxinB in Clinical Trials for Parkinson's disease

Protocol

March 31, 2016

Prepared by:

Ivy Medical Communications

TABLE OF CONTENTS

1 PROJECT OVERVIEW 2

2 LITERATURE SEARCH STRATEGY 2

2.1 Medical Literature Search Strategy 2

3 INCLUSION AND EXCLUSION CRITERIA 14

3.1 Study Selection Process 17

4 DATA EXTRACTION AND REPORTING OF RESULTS 19

4.1 Variable Headings for Evidence Tables 20

4.2 Evidence Table Templates 20

4.3 Reporting of Results 20

5 REFERENCES 20

APPENDIX A: CRITICAL APPRAISAL OF CLINICAL TRIAL STUDIES 22

PROJECT OVERVIEW

The primary objective of this project is to produce comprehensive evidenced-based data report that elucidates dosing practices and associated outcomes for RimabotulinumtoxinB . This systematic literature review focuses on identifying clinical trials, systematic reviews, and meta-analyses.

LITERATURE SEARCH STRATEGY

A literature search strategy based on the concepts and disease area(s) of interest was prepared, with the goal of identifying all relevant published literature within the search period. The literature search strategy used a combination of Medical Subject Heading (MeSH) terms and key words.

DATA SOURCES

The following medical literature databases were used to identify relevant articles for inclusion in the systematic literature review:

PubMed

Cochrane Library

Embase

Medical Literature Search Strategy

The following preliminary search strategy was conducted in PubMed on December, 2015. An additional search in the Cochrane Library and Embase was conducted as well. The search was intended to capture all RimabotulinumtoxinB clinical trials with the following conditions of interest:

- Cervical dystonia
- Torticollis
- Blepharospasm
- Limb dystonia
- Focal dystonia
- Generalized dystonia
- Segmental dystonia
- Oromandibular dystonia
- Jaw dystonia
- Bruxism
- Cranial dystonia
- Lingual dystonia

- Hemifacial spasm
- Facial myoclonus
- Facial synkinesis
- Spasmodic dysphonia
- Laryngeal spasm
- Spasticity
- Spastic paresis
- Myofascial spasm
- Cerebral palsy
- Writers' cramp
- Hand dystonia
- Foot dystonia
- Musicians dystonia
- Task specific focal dystonia
- Musician's cramp
- Embouchure dystonia
- Sialhorrhea
- Drooling
- Hypersalivation
- Seventh nerve facial disorders

0 presents search terms by category with the respective results identified in PubMed.

Limits for the search included the following:

Published in English

Published from January 1, 1999 to December 31, 2015

Preliminary PubMed Search Strategy

Search No.	Search Terms	No. of Results
**Drug terms**		
#1	"RimabotulinumtoxinB" OR "Rimabotulinumtoxin B" OR "rimabotulinum toxin B" OR "Myobloc" English, Publication date from 1999-2015	492
**Indication/health condition terms**		
#2	"Cervical Dystonia" Limits: English, Publication Date from 1999-2015	2065
#3	"Torticollis" Limits: English, Publication Date from 1999-2015	1582
#4	" Blepharospasm"[Text Word] Limits: English, Publication Date from 1999-2015	864
#5	"Limb dystonia"[Text Word] Limits: English, Publication Date from 1999-2015	3838
#6	"Focal dystonia "[Text Word] Limits: English, Publication Date from 1999-2015	638
#7	"Generalized dystonia"[Text Word] Limits: English, Publication Date from 1999-2015	232

#8	"Segmental dystonia" [Text Word] Limits: English, Publication Date from 1999-2015	167
#9	“Oromandibular dystonia” [Text Word] Limits: English, Publication Date from 1999-2015	122
#10	“Jaw dystonia” [Text Word] Limits: English, Publication Date from 1999-2015	1591
#11	“Bruxism” [Text Word] Limits: English, Publication Date from 1999-2015	291
#12	“Cranial Dystonia” [Text Word] Limits: English, Publication Date from 1999-2015	124
#13	“Lingual Dystonia” [Text Word] Limits: English, Publication Date from 1999-2015	836
#14	“Hemifacial Spasm” [Text Word] Limits: English, Publication Date from 1999-2015	127
#15	“Facial myoclonus” [Text Word] Limits: English, Publication Date from 1999-2015	227
#16	“Facial synkinesis ” [Text Word] Limits: English, Publication Date from 1999-2015	2318
#17	“Spasmodic dysphonia” [Text Word] Limits: English, Publication Date from 1999-2015	433
#18	“Laryngeal spasm” [Text Word] Limits: English, Publication Date from 1999-2015	6198
#19	“Spasticity” [Text Word] Limits: English, Publication Date from 1999-2015	663
#20	“Spastic paresis” [Text Word] Limits: English, Publication Date from 1999-2015	45
#21	“Myofascial spasm” [Text Word] Limits: English, Publication Date from 1999-2015	12698
#22	“Cerebral palsy” [Text Word] Limits: English, Publication Date from 1999-2015	3331
#23	“Writers’ cramp” [Text Word] Limits: English, Publication Date from 1999-2015	710
#24	“Hand dystonia” [Text Word] Limits: English, Publication Date from 1999-2015	23
#25	“Foot dystonia” [Text Word] Limits: English, Publication Date from 1999-2015	141
#26	“Musicians dystonia” [Text Word] Limits: English, Publication Date from 1999-2015	51
#27	“Task specific focal dystonia” [Text Word] Limits: English, Publication Date from 1999-2015	22
#28	“Musician’s cramp” [Text Word] Limits: English, Publication Date from 1999-2015	16
#29	“Embouchure dystonia” [Text Word] Limits: English, Publication Date from 1999-2015	763

#30	“Sialhorrhea” [Text Word] Limits: English, Publication Date from 1999-2015	1064
#31	“Drooling” [Text Word] Limits: English, Publication Date from 1999-2015	929
#32	“Hypersalivation” [Text Word] Limits: English, Publication Date from 1999-2015	48
#33	“Seventh nerve facial disorders” [Text Word] Limits: English, Publication Date from 1999-2015	2065
#34	“Cervical dystonia"[Text Word] OR “Torticollis"[Text Word] OR Blepharospasm"[Text Word] OR “Limb dystonia"[Text Word] OR “Focal dystonia"[Text Word] OR “Generalized dystonia"[Text Word] OR “Segmental dystonia"[Text Word] OR “Oromandibular dystonia"[Text Word] OR “Jaw dystonia"[Text Word] OR “Bruxism"[Text Word] OR “Cranial dystonia"[Text Word] OR “Lingual dystonia"[Text Word] OR “Hemifacial spasm"[Text Word] OR “Facial myoclonus"[Text Word] OR “Facial synkinesis"[Text Word] OR “Spasmodic dysphonia"[Text Word] OR “Laryngeal spasm"[Text Word] OR Spasticity"[Text Word] OR “Spastic paresis"[Text Word] OR “Myofascial spasm"[Text Word] OR “Cerebral palsy"[Text Word] OR “Writers’ cramp "[Text Word] OR “Hand dystonia"[Text Word] OR “Foot dystonia"[Text Word] OR “Musicians dystonia” OR “Task specific focal dystonia"[Text Word] OR “Musician’s cramp"[Text Word] OR “Embouchure dystonia"[Text Word] OR “Sialhorrhea"[Text Word] OR “Drooling"[Text Word] OR “Hypersalivation"[Text Word] OR"[Text Word] “Seventh nerve facial disorders"[Text Word] Limits: English, Publication Date from 1999-2015	32,867
#35	#1 AND #34 AND Limits: English, Publication Date from Publication Date from 1999-2015	**490**
#36	#1 AND #2 Limits: English, Publication Date from 1999-2015	**99**
#37	#1 AND #3 AND Limits: English, Publication Date from 1999-2015	**34**
#38	#1 AND #4 Limits: English, Publication Date from 1999-2015	**24**
#39	#1 AND #5 Limits: English, Publication Date from 1999-2015	**33**
#40	#1 AND #6 Limits: English, Publication Date from 1999-2015	**43**
#41	#1 AND #7 Limits: English, Publication Date from 1999-2015	**1**
#42	#1 AND #8 Limits: English, Publication Date from 1999-2015	**0**
#43	#1 AND #9 Limits: English, Publication Date from 1999-2015	**2**
#44	#1 AND #10 Limits: English, Publication Date from 1999-2015	**0**

#45	#1 AND #11 Limits: English, Publication Date from 1999-2015	**2**
#46	#1 AND #12 Limits: English, Publication Date from 1999-2015	**0**
#47	#1 AND #13 Limits: English, Publication Date from 1999-2015	**0**
#48	#1 AND #14 Limits: English, Publication Date from 1999-2015	**13**
#49	#1 AND #15 Limits: English, Publication Date from 1999-2015	**0**
#50	#1 AND #16 Limits: English, Publication Date from 1999-2015	**0**
#51	#1 AND #17 Limits: English, Publication Date from 1999-2015	**4**
#52	#1 AND #18 Limits: English, Publication Date from 1999-2015	**2**
#53	#1 AND #19 Limits: English, Publication Date from 1999-2015	**44**
#54	#1 AND #20 Limits: English, Publication Date from 1999-2015	**3**
#55	#1 AND #21 Limits: English, Publication Date from 1999-2015	**0**
#56	#1 AND #22 Limits: English, Publication Date from 1999-2015	**15**
#57	#1 AND #23 Limits: English, Publication Date from 1999-2015	**55**
#58	#1 AND #24 Limits: English, Publication Date from 1999-2015	**7**
#59	#1 AND #25 Limits: English, Publication Date from 1999-2015	**2**
#60	#1 AND #26 Limits: English, Publication Date from 1999-2015	**1**
#61	#1 AND #27 Limits: English, Publication Date from 1999-2015	**0**
#62	#1 AND #28 Limits: English, Publication Date from 1999-2015	**0**
#63	#1 AND #29 Limits: English, Publication Date from 1999-2015	**0**
#64	#1 AND #30 Limits: English, Publication Date from 1999-2015	**40**
#65	#1 AND #31 Limits: English, Publication Date from 1999-2015	**31**
#66	#1 AND #32 Limits: English, Publication Date from 1999-2015	**36**
#67	#1 AND #33 Limits: English, Publication Date from 1999-2015	**0**

0 presents summarized literature search results for all conditions. There were 285 unique records identified across conditions. Unique records were also identified for each condition (cervical dystonia, blepharospam, hemifacial spasm, spasticity, writer's cramp, sialorrhea and hypersalivation) are presented in 0 through Table 34.

Summary of Literature Search Results for All Conditions

Database	Records	Unique Records
PubMed	362	27
Cochrane	24	4
Embase	104	3
**Totals**	**490**	**34**

Summary of Literature Search Results for Cervical dystonia

Database	Records	Unique Records
PubMed	73	5
Cochrane	5	1
Embase	21	1
**Totals**	**99**	**7**

Summary of Literature Search Results for Torticollis

Database	Records	Unique Records
PubMed	19	0
Cochrane	4	0
Embase	11	0
**Totals**	**34**	**0**

N/A = Not available

Summary of Literature Search Results for Blepharospasm

Database	Records	Unique Records
PubMed	10	1
Cochrane	2	1
Embase	12	1
**Totals**	**24**	**3**

Summary of Literature Search Results for Limb dystonia

Database	Records	Unique Records
PubMed	29	0
Cochrane	0	0
Embase	4	0
**Totals**	**33**	**0**

Table 7. Summary of Literature Search Results for Focal dystonia

Database	Records	Unique Records
PubMed	31	0
Cochrane	5	0
Embase	7	0
**Totals**	**43**	**0**

Table 8. Summary of Literature Search Results for Generalized dystonia

Database	Records	Unique Records
PubMed	1	0
Cochrane	0	0
Embase	0	0
**Totals**	**1**	**0**

Table 9. Summary of Literature Search Results for Segmental dystonia

Database	Records	Unique Records
PubMed	0	0
Cochrane	0	0
Embase	0	0
**Totals**	**0**	**0**

Table 10. Summary of Literature Search Results for Oromandibular dystonia

Database	Records	Unique Records
PubMed	0	0
Cochrane	0	0
Embase	2	0
**Totals**	**2**	**0**

Table 11. Summary of Literature Search Results for Jaw dystonia

Database	Records	Unique Records
PubMed	0	0
Cochrane	0	0
Embase	0	0
**Totals**	**0**	**0**

Table 12. Summary of Literature Search Results for Bruxism

Database	Records	Unique Records
PubMed	2	0
Cochrane	0	0
Embase	0	0
**Totals**	**2**	**0**

Table 13. Summary of Literature Search Results for Cranial dystonia

Database	Records	Unique Records
PubMed	0	0
Cochrane	0	0
Embase	0	0
**Totals**	**0**	**0**

Table 14. Summary of Literature Search Results for Lingual dystonia

Database	Records	Unique Records
PubMed	0	0
Cochrane	0	0
Embase	0	0
**Totals**	**0**	**0**

Table 15. Summary of Literature Search Results for Hemifacial spasm

Database	Records	Unique Records
PubMed	7	0
Cochrane	0	0
Embase	6	0
**Totals**	**13**	**0**

Table 16. Summary of Literature Search Results for Facial myoclonus

Database	Records	Unique Records
PubMed	0	0
Cochrane	0	0
Embase	0	0
**Totals**	**0**	**0**

Table 17. Summary of Literature Search Results for Facial synkinesis

Database	Records	Unique Records
PubMed	0	0
Cochrane	0	0
Embase	0	0
**Totals**	**0**	**0**

Table 18. Summary of Literature Search Results for Spasmodic dysphonia

Database	Records	Unique Records
PubMed	2	0
Cochrane	1	0
Embase	1	0

**Totals**	**4**	**0**

Table 19. Summary of Literature Search Results for Laryngeal spasm

Database	Records	Unique Records
PubMed	1	0
Cochrane	0	0
Embase	1	0
**Totals**	**2**	**0**

Table 20. Summary of Literature Search Results for Spasticity

Database	Records	Unique Records
PubMed	31	3
Cochrane	2	1
Embase	11	1
**Totals**	**44**	**5**

Table 21. Summary of Literature Search Results for Spastic paresis

Database	Records	Unique Records
PubMed	3	0
Cochrane	0	0
Embase	0	0
**Totals**	**3**	**0**

Table 22. Summary of Literature Search Results for Myofascial spasm

Database	Records	Unique Records
PubMed	0	0
Cochrane	0	0
Embase	0	0
**Totals**	**0**	**0**

Table 23. Summary of Literature Search Results for Cerebral palsy

Database	Records	Unique Records
PubMed	13	0
Cochrane	1	0
Embase	1	0

**Totals**	**15**	**0**

Table 24. Summary of Literature Search Results for Writers' cramp

Database	Records	Unique Records
PubMed	54	5
Cochrane	0	0
Embase	1	0
**Totals**	**51**	**5**

Table 25. Summary of Literature Search Results for Hand dystonia

Database	Records	Unique Records
PubMed	3	0
Cochrane	0	0
Embase	4	0
**Totals**	**7**	**0**

Table 26. Summary of Literature Search Results for Foot dystonia

Database	Records	Unique Records
PubMed	1	0
Cochrane	1	0
Embase	0	0
**Totals**	**2**	**0**

Table 27. Summary of Literature Search Results for Musicians dystonia

Database	Records	Unique Records
PubMed	1	0
Cochrane	0	0
Embase	0	0
**Totals**	**0**	**0**

Table 28. Summary of Literature Search Results for Task specific focal dystonia

Database	Records	Unique Records
PubMed	0	0
Cochrane	0	0
Embase	0	0

**Totals**	**0**	**0**

Table 29. Summary of Literature Search Results for Musician's cramp

Database	Records	Unique Records
PubMed	0	0
Cochrane	0	0
Embase	0	0
**Totals**	**0**	**0**

Table 30. Summary of Literature Search Results for Embouchure dystonia

Database	Records	Unique Records
PubMed	0	0
Cochrane	0	0
Embase	0	0
**Totals**	**0**	**0**

Table 31. Summary of Literature Search Results for Sialhorrhea

Database	Records	Unique Records
PubMed	27	6
Cochrane	2	1
Embase	11	0
**Totals**	**40**	**7**

Table 32. Summary of Literature Search Results for Drooling

Database	Records	Unique Records
PubMed	27	5
Cochrane	1	1
Embase	3	0
**Totals**	**31**	**6**

Table 33. Summary of Literature Search Results for Hypersalivation

Database	Records	Unique Records
PubMed	27	6
Cochrane	0	0
Embase	8	0

Totals	35	6

Table 34. Summary of Literature Search Results for Seventh nerve facial disorders

Database	Records	Unique Records
PubMed	0	0
Cochrane	0	0
Embase	0	0
**Totals**	**0**	**0**

INCLUSION AND EXCLUSION CRITERIA

The inclusion and exclusion criteria are based on a strategy that will identify study types of interest within the population or disease condition of interest. The criteria listed in Table 35 and 36 will be applied to the 37 unique titles and abstracts identified by the medical literature search in Section 2.

Table 35. Level 1 Screening Criteria for Inclusion and Exclusion of Clinical Studies

Criteria	Included	Excluded
Study design	Randomized controlled clinical trials (phase 2 and phase 3)<br>Non–randomized controlled clinical trials (phase 2 and phase 3)<br>Long-term follow-up studies (e.g., open-label follow-up of randomized controlled clinical trials)	Preclinical<br>Phase 1<br>Prognostic/biomarker<br>Genetic<br>Retrospective<br>Registry<br>Case report<br>Non-comparative studies<br>Letters<br>Consensus reports<br>Editorials<br>Nonsystematic reviews<br>Systematic reviews and meta-analyses will not be included in their own right; they will be used for identification of additional primary studies.

Criteria	Included	Excluded
Population	Patients with the following conditions:<br>Cervical dystonia<br>Torticollis<br>Blepharospasm<br>Limb dystonia<br>Focal dystonia<br>Generalized dystonia<br>Segmental dystonia<br>Oromandibular dystonia<br>Jaw dystonia<br>Bruxism<br>Cranial dystonia<br>Lingual dystonia<br>Hemifacial spasm<br>Facial myoclonus<br>Facial synkinesis<br>Spasmodic dysphonia<br>Laryngeal spasm<br>Spasticity<br>Spastic paresis<br>Myofascial spasm<br>Cerebral palsy<br>Writers' cramp<br>HOR dystonia<br>Foot dystonia<br>Musicians dystonia<br>Task specific focal dystonia<br>Musician's cramp<br>Embouchure dystonia<br>Sialhorrhea<br>Drooling<br>Hypersalivation<br>Seventh nerve facial disorders	
Interventions	RimabotulinumtoxinB	All studies that do not include the intervention RimabotulinumtoxinB, myobloc
Outcomes	Study methodology, patient, and treatment level data	Biochemical or immunologic endpoints

[a]Systematic reviews and meta-analyses will not be included in their own right, but will be used for identification of primary studies.

Table 36. Level 2 Screening Criteria for Inclusion and Exclusion of Clinical Studies

Criteria	Included	Excluded
	Criteria in Table 35	Only biochemical or immunologic endpoints will be excluded.<br>Nonrandomized controlled phase 2 or 3 clinical trials<br>Comparative long-term follow-up studies<br>Open-label follow-up of randomized controlled clinical trials<br>Comparative prospective phase 4
Population	Criteria in Table 35	Criteria in Table 35<br>No further restriction
Interventions	Criteria in Table 35	Criteria in Table 35<br>No further restriction
Outcomes	Primary and secondary endpoints for:<br>○ Safety (include all dosing-related parameters)<br>○ Efficacy<br>(Patient-reported and Economic [if available])<br>Tertiary endpoints	No further restriction

For studies excluded at the level 2 selection process, the study citations will be tabulated and the reason for exclusion of individual studies will be recorded.

Included studies will be categorized as randomized controlled trials (RCTs), non-RCTs, comparative long-term follow-up clinical trials, and comparative prospective observational studies. Randomized controlled trials will be advanced to the formal data extraction and quality assessment process. If the number of RCTs identified is less than 25 studies, other study types (non-RCTs and observational studies) will be evaluated for formal data extraction and quality assessment.

Study Selection Process

At level 1 screening, titles and abstracts of the identified studies from the electronic databases and Internet searches will be scanned by two researchers independently to evaluate the potential study relevance using the criteria outlined in Table 35 and Table 36. If there is uncertainty regarding the relevance of the study, the two researchers will review and reconcile discrepancies to reach consensus. Bibliographic reference lists of systematic reviews identified during level 1 screening will be searched to identify any relevant studies that were not identified through the electronic database searches.

At level 2 screening, full texts of studies selected at level 1 will be reviewed, and the same inclusion and exclusion criteria will be applied.

The inclusion and exclusion processes will be thoroughly documented, including completion of the Preferred Reporting Items for Systematic Reviews and Meta-Analyses (PRISMA) flow diagram (Moher et al., 2009) for studies included at the level 2 screening. For studies excluded at level 1, the reason for exclusion will be recorded and the number of studies excluded for each reason summarized. For studies excluded at level 2, the study citations will be tabulated and the reason for exclusion of individual studies will also be recorded.

PRISMA Diagram

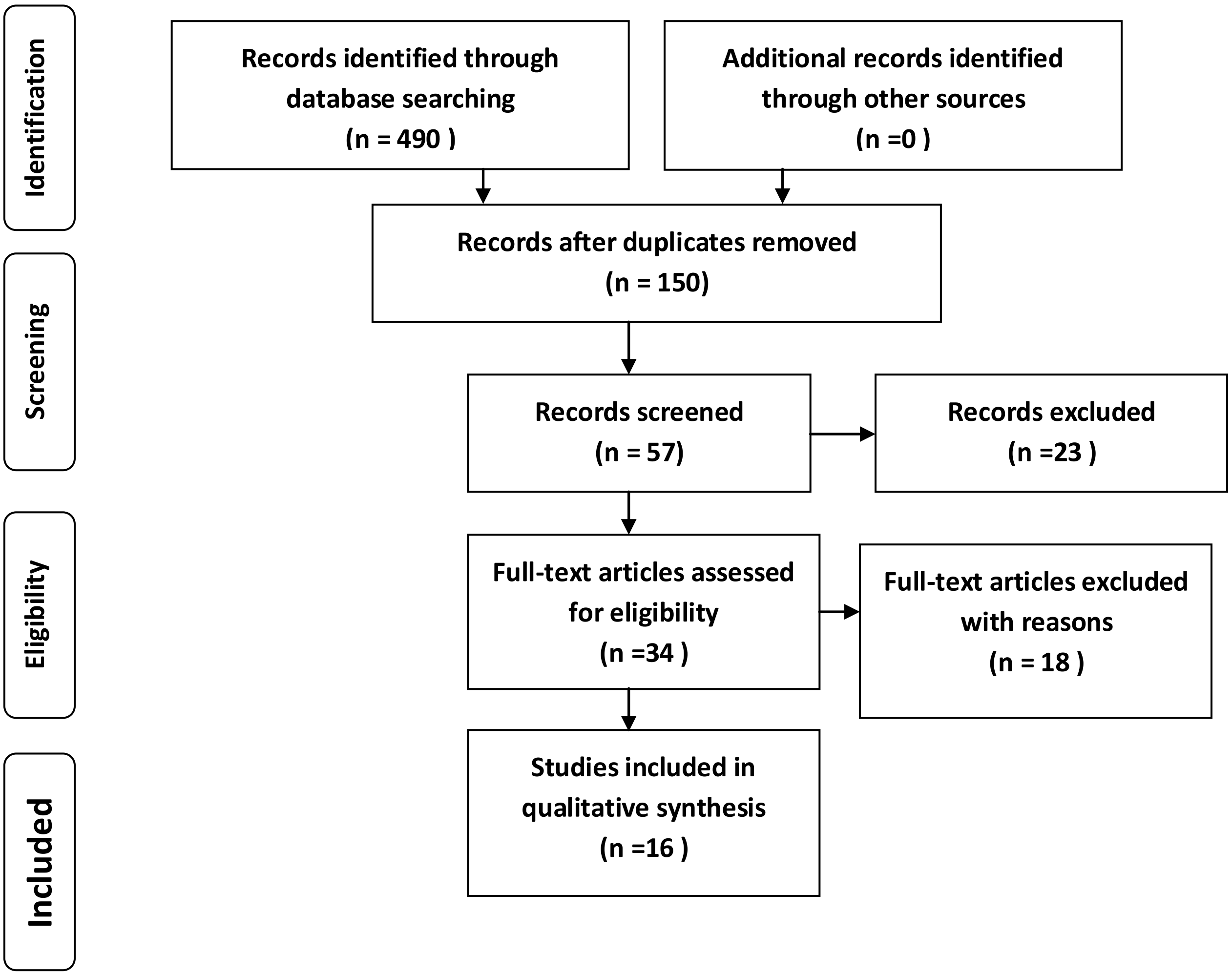

DATA EXTRACTION AND REPORTING OF RESULTS

Data will be extracted for the outcomes of interest for all relevant time points for all RCTs. If fewer than 25 RCTs are identified, non-RCTs and other studies will be evaluated for data extraction.

The relevant data will be extracted into evidence tables in Microsoft Word. All data-extraction templates will be created and agreed upon by the project team before commencing data extraction.

Data will be extracted from full-text versions of studies where available (i.e., we will not use abstracts unless an abstract is the terminal source document). Quality-control procedures for

the data extraction will comprise verification of all extracted data with their original sources by a second researcher.

Variable Headings for Evidence Tables

Proposed column headings for each study may include the following:

Study identifiers (author[s], date, study acronym, sponsors, quality assessment)

Study design (type, location, brief details of study design, inclusion/exclusion criteria, patient age groups, sample size)

Treatment (drug therapy, best supportive care, minimally invasive procedure, and/or placebo)

Outcomes (efficacy, safety, other [patient-reported outcomes, economic])

Evidence Table Templates

The data extraction outline will include all draft evidence table templates.

Reporting of Results

In lieu of a formal final study report, the research team will prepare a final data extraction summary of tables.

The final data extraction outline will present table templates and elements for extraction.

REFERENCES

Des Jarlais DC, Lyles C, Crepaz N.TREND Group. Improving the reporting quality of nonrandomized evaluations of behavioral and public health interventions: the TREND statement. Am J Public Health. 2004 Mar;94(3):361-6.

Higgins JPT, Green S (editors). Cochrane Handbook for Systematic Reviews of Interventions Version 5.1.0 [updated March 2011]. The Cochrane Collaboration, 2011. Available from www.cochrane-handbook.org.

Moher D, Liberati A, Tetzlaff J, Altman DG; PRISMA Group. Preferred reporting items for systematic reviews and meta-analyses: the PRISMA statement. J Clin Epidemiol. 2009 Oct;62(10):1006-12.

National Health Service. Public Health Resource Unit. Critical Appraisal Skills Program (CASP): cohort studies. 2004. Available at: http://www.unisa.edu.au/cahe/Resources/CAT/default.asp#Non_RCT. Accessed August 15, 2011.

National Institute for Health and Clinical Excellence (NICE). Single Technology Appraisal: specification for manufacturer/sponsor submission of evidence. October 2009. Available at: http://www.nice.org.uk/media/D6D/E2/SpecificationForManufacturerSponsorSubmissionEvidence.doc. Accessed August 15, 2011.

Appendix A:
Critical Appraisal of Clinical Trial Studies

Cochrane Quality Assessment Template for Clinical Studies

Domain	Support for Judgment<br><br>(Study specific information entered here)	Author's Judgment on 'Risk of Bias'<br><br>High, Low, Unclear
Selection bias.		
**Random sequence generation.**	Describe the method used to generate the allocation sequence in sufficient detail to allow an assessment of whether it should produce comparable groups.	Selection bias (biased allocation to interventions) due to inadequate generation of a randomised sequence.
**Allocation concealment.**	Describe the method used to conceal the allocation sequence in sufficient detail to determine whether intervention allocations could have been foreseen in advance of, or during, enrolment.	Selection bias (biased allocation to interventions) due to inadequate concealment of allocations prior to assignment.
Performance bias.		
**Blinding of participants and personnel** *Assessments should be made for each main outcome (or class of outcomes).*	Describe all measures used, if any, to blind study participants and personnel from knowledge of which intervention a participant received. Provide any information relating to whether the intended blinding was effective.	Performance bias due to knowledge of the allocated interventions by participants and personnel during the study.
Detection bias.		
**Blinding of outcome assessment** *Assessments should be made for each main outcome (or class of outcomes)*.	Describe all measures used, if any, to blind outcome assessors from knowledge of which intervention a participant received. Provide any information relating to whether the intended blinding was effective.	Detection bias due to knowledge of the allocated interventions by outcome assessors.
Attrition bias.		

Domain	Support for Judgment<br><br>(Study specific information entered here)	Author's Judgment on 'Risk of Bias'<br><br>High, Low, Unclear
**Incomplete outcome data** *Assessments should be made for each main outcome (or class of outcomes).*	Describe the completeness of outcome data for each main outcome, including attrition and exclusions from the analysis. State whether attrition and exclusions were reported, the numbers in each intervention group (compared with total randomized participants), reasons for attrition/exclusions where reported, and any re-inclusions in analyses performed by the review authors.	Attrition bias due to amount, nature or handling of incomplete outcome data.

Reporting bias.		
**Selective reporting.**	State how the possibility of selective outcome reporting was examined by the review authors, and what was found.	Reporting bias due to selective outcome reporting.
Other bias		
**Other sources of bias.**	State any important concerns about bias not addressed in the other domains in the tool. If particular questions/entries were pre-specified in the review's protocol, responses should be provided for each question/entry.	Bias due to problems not

*See Table A-2 for criteria to assess 'Risk of bias'

Adapted from Higgins JPT, Green S (editors). Cochrane Handbook for Systematic Reviews of Interventions Version 5.1.0 [updated March 2011]. The Cochrane Collaboration, 2011, Chapter 8.5.a. Available at http://www.mrc-bsu.cam.ac.uk/cochrane/handbook/. Accessed Novemeber 1, 2011.

Cochrane Criteria for Judging Risk of Bias

<table>
<tr><th colspan="2">RANDOM SEQUENCE GENERATION

Selection bias (biased allocation to interventions) due to inadequate generation of a randomised sequence.</th></tr>
<tr><td>Criteria for a judgment of ‘Low risk’ of bias.</td><td>The investigators describe a random component in the sequence generation process such as:
Referring to a random number table;
Using a computer random number generator;
Coin tossing;
Shuffling cards or envelopes;
Throwing dice;
Drawing of lots;
Minimization*.
*Minimization may be implemented without a random element, and this is considered to be equivalent to being random.</td></tr>
<tr><td>Criteria for the judgment of ‘High risk’ of bias.</td><td>The investigators describe a non-random component in the sequence generation process. Usually, the description would involve some systematic, non-random approach, for example:
Sequence generated by odd or even date of birth;
Sequence generated by some rule based on date (or day) of admission;
Sequence generated by some rule based on hospital or clinic record number.
Other non-random approaches happen much less frequently than the systematic approaches mentioned above and tend to be obvious. They usually involve judgement or some method of non-random categorization of participants, for example:
Allocation by judgement of the clinician;
Allocation by preference of the participant;
Allocation based on the results of a laboratory test or a series of tests;
Allocation by availability of the intervention.</td></tr>
<tr><td>Criteria for the judgment of ‘Unclear risk’ of bias.</td><td>Insufficient information to permit judgement of ‘Low risk’ or ‘High risk’. This is usually the case if the method of concealment is not described or not described in sufficient detail to allow a definite judgement – for example if the use of assignment envelopes is described, but it remains unclear whether envelopes were sequentially numbered, opaque and sealed.</td></tr>
</table>

BLINDING OF PARTICIPANTS AND PERSONNEL<br>**Performance bias due to knowledge of the allocated interventions by participants and personnel during the study.**	
Criteria for a judgment of 'Low risk' of bias.	Any one of the following:<br>• No blinding or incomplete blinding, but the review authors judge that the outcome is not likely to be influenced by lack of blinding;<br>Blinding of participants and key study personnel ensured, and unlikely that the blinding could have been broken.
Criteria for the judgment of 'High risk' of bias.	Any one of the following:<br>• No blinding or incomplete blinding, and the outcome is likely to be influenced by lack of blinding;<br>Blinding of key study participants and personnel attempted, but likely that the blinding could have been broken, and the outcome is likely to be influenced by lack of blinding.
Criteria for the judgment of 'Unclear risk' of bias.	Any one of the following:<br>• Insufficient information to permit judgment of 'Low risk' or 'High risk';<br>The study did not address this outcome.

INCOMPLETE OUTCOME DATA<br>**Attrition bias due to amount, nature or handling of incomplete outcome data.**	
Criteria for a judgment of 'Low risk' of bias.	Any one of the following:<br>• No missing outcome data;<br>• Reasons for missing outcome data unlikely to be related to true outcome (for survival data, censoring unlikely to be introducing bias);<br>• Missing outcome data balanced in numbers across intervention groups, with similar reasons for missing data across groups;<br>• For dichotomous outcome data, the proportion of missing outcomes compared with observed event risk not enough to have a clinically relevant impact on the intervention effect estimate;<br>• For continuous outcome data, plausible effect size (difference in means or standardized difference in means) among missing outcomes not enough to have a clinically relevant impact on observed effect size;<br>Missing data have been imputed using appropriate methods.
Criteria for the judgment of 'High risk' of bias.	Any one of the following:<br>• Reason for missing outcome data likely to be related to true outcome, with either imbalance in numbers or reasons for missing data across intervention groups;<br>• For dichotomous outcome data, the proportion of missing outcomes compared with observed event risk enough to induce clinically relevant bias in intervention effect estimate;<br>• For continuous outcome data, plausible effect size (difference in means or standardized difference in means) among missing outcomes enough to induce clinically relevant bias in observed effect size;<br>• 'As-treated' analysis done with substantial departure of the intervention received from that assigned at randomization;<br>Potentially inappropriate application of simple imputation.
Criteria for the judgment of 'Unclear risk' of bias.	Any one of the following:<br>• Insufficient reporting of attrition/exclusions to permit judgment of 'Low risk' or 'High risk' (e.g. number randomized not stated, no reasons for missing data provided);<br>The study did not address this outcome.

**SELECTIVE REPORTING**<br>**Reporting bias due to selective outcome reporting.**	
Criteria for a judgment of 'Low risk' of bias.	Any of the following:<br>• The study protocol is available and all of the study's pre-specified (primary and secondary) outcomes that are of interest in the review have been reported in the pre-specified way;<br>The study protocol is not available but it is clear that the published reports include all expected outcomes, including those that were pre-specified (convincing text of this nature may be uncommon).
Criteria for the judgment of 'High risk' of bias.	Any one of the following:<br>• Not all of the study's pre-specified primary outcomes have been reported;<br>• One or more primary outcomes is reported using measurements, analysis methods or subsets of the data (e.g. subscales) that were not pre-specified;<br>• One or more reported primary outcomes were not pre-specified (unless clear justification for their reporting is provided, such as an unexpected adverse effect);<br>• One or more outcomes of interest in the review are reported incompletely so that they cannot be entered in a meta-analysis;<br>The study report fails to include results for a key outcome that would be expected to have been reported for such a study.
Criteria for the judgment of 'Unclear risk' of bias.	Insufficient information to permit judgment of 'Low risk' or 'High risk'. It is likely that the majority of studies will fall into this category.
**OTHER BIAS**<br>**Bias due to problems not covered elsewhere in the table.**	
Criteria for a judgment of 'Low risk' of bias.	The study appears to be free of other sources of bias.
Criteria for the judgment of 'High risk' of bias.	There is at least one important risk of bias. For example, the study:<br>• Had a potential source of bias related to the specific study design used; or<br>• Has been claimed to have been fraudulent; or<br>Had some other problem.
Criteria for the judgment of 'Unclear risk' of bias.	There may be a risk of bias, but there is either:<br>• Insufficient information to assess whether an important risk of bias exists; or<br>Insufficient rationale or evidence that an identified problem will introduce bias.

Adapted from Higgins JPT, Green S (editors). Cochrane Handbook for Systematic Reviews of Interventions Version 5.1.0 [updated March 2011]. The Cochrane Collaboration, 2011, Chapter 8.5.d. Available at http://www.mrc-bsu.cam.ac.uk/cochrane/handbook/. Accessed Novemeber 1, 2011.
